# Acute effects of short-term high-intensity interval exercise and moderate-intensity aerobic exercise on food-related inhibitory control in obese adults: a randomized controlled crossover trial using ERP measures

**DOI:** 10.3389/fpsyg.2025.1634569

**Published:** 2025-07-23

**Authors:** Bo Sun, Yun-Fei Lu, Ji-Wei Chen, Yi-Lun Xiao, Jie Ren

**Affiliations:** ^1^School of Athletic Performance, Shanghai University of Sport, Shanghai, China; ^2^China Table Tennis College, Shanghai University of Sport, Shanghai, China; ^3^School of Physical Education, Shanghai University of Sport, Shanghai, China; ^4^School of Humanities and Social Sciences, Harbin Institute of Technology (Shenzhen), Shenzhen, China

**Keywords:** acute exercise, inhibitory function, gender difference, ERP, HIIE, obesity

## Abstract

**Introduction:**

Food-specific inhibitory control plays a critical role in maintaining a healthy body weight. However, limited research has explored how different exercise modalities influence this form of control in adults with obesity, particularly regarding the underlying neural mechanisms. This study aimed to examine the acute effects of short-term high-intensity interval exercise (HIIE) and moderate-intensity aerobic exercise (MIAE) on food-related inhibitory function in obese adults, and to assess whether sex differences modulate the response to exercise interventions. The findings aim to provide evidence-based guidance for the personalized design of exercise prescriptions targeting dietary behavior regulation in this population.

**Methods:**

A total of 32 obese adults participated in a within-subjects randomized crossover design. Each individual completed three separate sessions: (1) 15 min of HIIE on a power-adjusted cycle ergometer, (2) 30 min of MIAE, and (3) a 30-min resting control condition. After each session, participants performed a food-related Go/NoGo task during which behavioral responses (reaction time and accuracy) and event-related potential (ERP) components (N2 and P3 amplitudes) were recorded.

**Results:**

Across all image types, both male and female participants demonstrated shorter reaction times following HIIE and MIAE compared to the control condition. In males, reaction times were tended to be shorter under HIIE than under MIAE, although no significant differences in accuracy were observed across conditions. Additionally, female participants showed enhanced N2 amplitudes in NoGo trials involving low-calorie food images under the HIIE condition, and no significant difference between NoGo and Go P3 amplitudes when responding to high-calorie food stimuli.

**Conclusion:**

(1) HIIE may enhance behavioral response speed in obese males through non-inhibitory optimization of the prefrontal–striatal pathway, reflecting the neural efficiency hypothesis associated with short-term exercise; (2) MIAE may improve conflict monitoring in obese females, facilitating a shift in inhibitory control over high-calorie foods from active suppression to automated processing. These findings highlight the importance of tailoring food inhibition interventions to account for exercise intensity adaptability and sex-specific neuro-metabolic targets, providing a scientific rationale for personalized exercise prescription. Future studies should further investigate the causal mechanisms through which HIIE modulates food-related inhibition and explore neuroregulatory targets for optimizing exercise-based interventions.

## 1 Introduction

In recent years, the prevalence of obesity has become increasingly severe. Research suggests that by 2030, the global rate of overweight and obesity among adults is expected to reach 58%, making it a major public health concern of the 21st century (Kelly et al., 2008). Obesity is associated with a range of physiological conditions, including diabetes, hypertension, and coronary heart disease, contributing to higher morbidity and all-cause mortality rates, and posing a substantial economic burden on society (Friedemann et al., 2012). More concerning is the potential detrimental impact of obesity on cognitive functioning (Miller and Spencer, 2014). Regarding the causes of overweight and obesity, Wood et al. emphasize the critical role of eating behaviors and food-related decision-making (Wood et al., 2016), particularly the excessive intake of high-calorie foods, as key factors contributing to weight gain. For example, the weight gain observed in individuals with obesity has been associated with an automatic approach tendency toward food (Carnell and Wardle, 2008), as well as heightened attentional focus on food cues (Guo et al., 2024).

Inhibitory control refers to the ability to suppress dominant or automatic responses, involving the regulation of attention, behavior, thoughts, or emotions in the face of strong internal or external stimuli. This ability facilitates goal-directed behavior and is considered a core subcomponent of executive function (Crone, 2009; Diamond, 2013). Neuroscientific evidence has shown that individuals with obesity exhibit systematic neurocognitive deficits in executive functioning, with impairments in inhibitory control being particularly pronounced (Yang et al., 2018). These deficits result in a tendency toward impulsive choices during food-related decision-making, characterized by enhanced attentional capture by high-calorie food cues (Do et al., 2024).

From the perspective of neuroplasticity induced by exercise interventions, enhancing inhibitory control in response to food-related stimuli has emerged as a novel target in obesity management (Smith et al., 2011; Miguet et al., 2023; Moschonis and Trakman, 2023). Existing literature suggests that acute exercise has a small-to-moderate positive effect on inhibitory function (Kempton et al., 2011; Chang et al., 2012; Guiney and Machado, 2013; Ludyga et al., 2016; Kao et al., 2018; Ligeza et al., 2018; Yang et al., 2018). However, some studies have reported null effects of acute exercise on inhibitory control (Lambourne and Tomporowski, 2010). Such inconsistencies may be attributed to variations in the intensity and duration of the exercise protocols used across studies (Olson et al., 2016; Wohlwend et al., 2017). Event-related potentials (ERPs) provide a useful tool for examining the neural underpinnings of inhibitory control. The N2 component, occurring approximately 200–350 ms after stimulus onset, is sensitive to conflict detection, with its amplitude increasing as inhibitory demands rise (Folstein and Van Petten, 2008; Larson et al., 2014). The P3 component, typically observed between 300 and 600 ms post-stimulus, is associated with attentional allocation and the engagement of inhibitory resources (Falkenstein et al., 1991; Gajewski and Falkenstein, 2013). In food-specific inhibitory control tasks, individuals with obesity exhibit a pronounced attentional bias toward food stimuli, particularly high-calorie foods. This is evidenced by significantly greater N2 amplitudes in response to food-related images compared to neutral stimuli, suggesting enhanced early attentional resource allocation to high-calorie food cues. Moreover, gender differences have been reported in the neural correlates of food-related cognitive control (Clayson et al., 2011; Melynyte et al., 2017). Specifically, obese females tend to rely more on the prefrontal-insular connectivity for inhibitory regulation, whereas obese males appear to engage the dorsolateral prefrontal–striatal pathways associated with reward processing. Compared to individuals with normal weight, those with obesity typically show attenuated N2 amplitudes and prolonged N2 latencies, indicating deficits in early conflict monitoring, which may compromise subsequent inhibitory control processes. Regarding the P3 component, studies have found that high-calorie food stimuli elicit smaller P3 amplitudes and longer latencies during NoGo trials in individuals with obesity relative to normal-weight counterparts, reflecting reduced recruitment of cognitive resources necessary for inhibition. Additionally, under resting conditions, P3 amplitudes during high-calorie food NoGo trials are significantly greater than those in Go trials, suggesting that inhibiting responses to high-calorie foods demands greater cognitive effort.

Recent studies have demonstrated that acute bouts of moderate-intensity aerobic exercise (MIAE) can indirectly influence eating behaviors by enhancing prefrontal cortical circuits related to inhibitory control, thereby reducing impulsive consumption of high-calorie foods (Carbine et al., 2024). This effect is reflected in increased N2 and P3 amplitudes in response to high-calorie food images. Notably, although traditional physical activity guidelines emphasize MIAE for promoting health benefits, adherence among adults with obesity tends to be low, which presents practical challenges and limits its feasibility in real-world applications (Bull et al., 2020). In addition to MIAE, high-intensity interval exercise (HIIE) has garnered considerable attention and preference, largely due to its “time-efficiency” (Chen and Zhu, 2024; Chen et al., 2025). Research has shown that HIIE may modulate eating behavior through lactate signaling, enhancing regulation along the hypothalamic–prefrontal pathway. Lactate enters the arcuate nucleus via monocarboxylate transporters, where it suppresses AgRP neuron activity and reduces the motivational drive to consume high-calorie foods (Jacob et al., 2023). Furthermore, lactate functions as a “metabolic buffer,” enhancing mitochondrial oxidative phosphorylation efficiency in the prefrontal cortex (PFC), thereby supporting more sustained inhibitory synaptic transmission (De Sousa Fernandes et al., 2022). These findings suggest the potential applicability of HIIE in enhancing food-specific inhibitory control.

Given that individuals with obesity differ significantly from those of normal weight in terms of metabolic status and responsiveness to exercise interventions (Van Galen et al., 2018), indirect evidence alone may be insufficient to formulate targeted recommendations for individuals with obesity. There is a clear need for direct empirical investigations into how exercise interventions modulate food-specific inhibitory control in this population. The aim of the present study is to investigate whether a single session of HIIE, compared to MIAE, serves as a time-efficient alternative—or potentially a superior strategy—for enhancing food-specific inhibitory control in obese adults. Furthermore, the study aims to explore whether the regulatory effects of these two exercise modalities differ by sex.

## 2 Materials and methods

### 2.1 Participants

All participants provided written informed consent prior to participation. The exclusion criteria included: current or past diagnosis of eating disorders or psychiatric conditions, history of head injury, body mass index (BMI) below the obesity threshold, and pregnancy or lactation at the time of the study. Eligible participants were adults within a specified age range who self-reported the ability to exercise at an intensity reaching 90% of their maximum heart rate (HRmax) for 16 min. Before enrollment, participants completed the Physical Activity Readiness Questionnaire (PAR-Q) (Arraiz et al., 1992) to assess their suitability for physical activity. Individuals who responded positively to any item on the PAR-Q were excluded from the study. Participants who completed all experimental sessions received appropriate compensation for their time and effort (Arraiz et al., 1992). Demographic characteristics of the participants are presented in Table 1.

**Table 1:** Basic information of participants.

Variables	Obses adults (*n* = 32)	
	Male (*n* = 16)	Female (*n* = 16)
Age (years)	24.32 ± 4.56	23.04 ± 5.52
Height (m)	173.24 ± 3.54	161.46 ± 2.55
Weight (kg)	95.85 ± 15.65	78.56 ± 10.45
BMI (kg/m^2^)	31.77 ± 3.27	30.11 ± 5.23

### 2.2 Experimental design

This study adopted a multifactorial mixed design. The between-subjects factor was gender (two levels: male and female), while the within-subjects factors included:

Exercise condition (three levels: resting control, high-intensity interval exercise [HIIE], and moderate-intensity aerobic exercise [MIAE]), trial type (two levels: Go and NoGo), image category (three levels: high-calorie food, low-calorie food, and neutral non-food), and electrode site (three levels: Fz, Cz, and Pz).

### 2.3 Measurements

Participants’ height and weight were measured to calculate body mass index (BMI). Height was measured using the SH-200 stadiometer (Shanghe, Zhengzhou, China), and weight was assessed with the Xiaomi S400 smart scale (Beijing, China).

The required sample size was calculated using G*Power 3.0, which indicated that a minimum of 28 participants was needed. This estimation was based on established parameters from prior studies: 80% statistical power, within-subject design, three-condition independent variable, and a significance level of *α* = 0.05 (Carbine et al., 2017; Ligeza et al., 2018). To account for potential dropouts during exercise intervention, a total of 32 obese adults were ultimately recruited for the study (Carbine et al., 2017; Ligeza et al., 2018).

### 2.4 Computerized task

**Figure 1:**
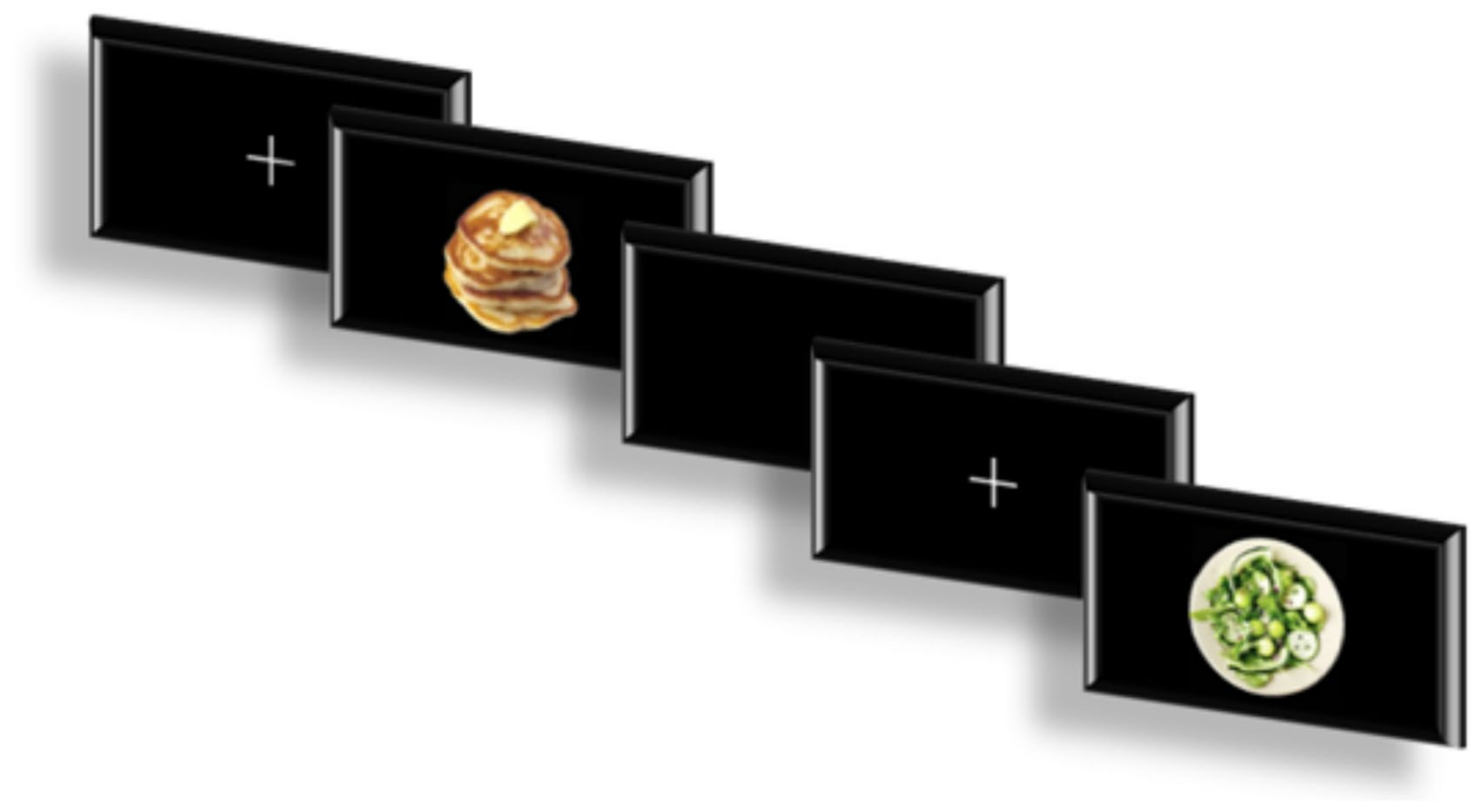
Flowchart of FoodGo/Nogo tasks.

The food-related Go/NoGo task employed in this study utilized image stimuli sourced from the standardized food-pics image database developed by Blechert (2014) which has been widely used in previous research (Killgore et al., 2013; Carbine et al., 2017). Based on caloric content, 60 high-calorie food images, 60 low-calorie food images, and 60 neutral non-food images were selected, for a total of 180 images. The structure of the Go/NoGo task followed the paradigm used by Bailey et al. and consisted of six distinct blocks presented in a randomized order: (1) High-calorie food as Go stimuli, neutral images as NoGo; (2) neutral images as Go, high-calorie food as NoGo; (3) high-calorie food as Go, low-calorie food as NoGo; (4) low-calorie food as Go, high-calorie food as NoGo; (5) low-calorie food as Go, neutral images as NoGo; (6) neutral images as Go, low-calorie food as NoGo (Bailey et al., 2021). Before the formal testing, participants completed a practice block consisting of 10 trials. In the formal session, the six task blocks were presented in random order. In each block, Go trials made up 75% of the trials and NoGo trials comprised 25%, ensuring participants developed a prepotent response tendency that required active inhibition on NoGo trials. A schematic representation of the task flow is shown in Figure 1.

### 2.5 Exercise program

The moderate-intensity aerobic exercise (MIAE) protocol was based on the studies by Kamijo et al. (2007) and O’Leary et al. (2011), as well as the latest guidelines from the American College of Sports Medicine (ACSM) (Thompson et al., 2013). The total duration was 30 min, consisting of a 2-min warm-up, followed by 28 min of cycling at 65–70% of individual HRmax, and ending with a cool-down phase. This duration and intensity align with meta-analytic findings on optimal exercise dosing for enhancing cognitive function (Chang et al., 2012).

The high-intensity interval exercise (HIIE) protocol was adapted from Tsukamoto et al. (2016) and Aulbach et al. (2020). Due to lower physiological tolerance and significantly reduced resting metabolic equivalents (METs) in obese adults, and based on pilot data indicating increased fatigue beyond 20 min, the total session duration was set to 16 min. The protocol included a 1-min warm-up, followed by six repetitions of 1.5-min high-intensity bouts at 80–90% HRmax, each separated by 1 min of active recovery at 50–65% HRmax. Maximum heart rate was calculated using the formula: HRmax = 207–0.7 × age.

**Figure 2:**
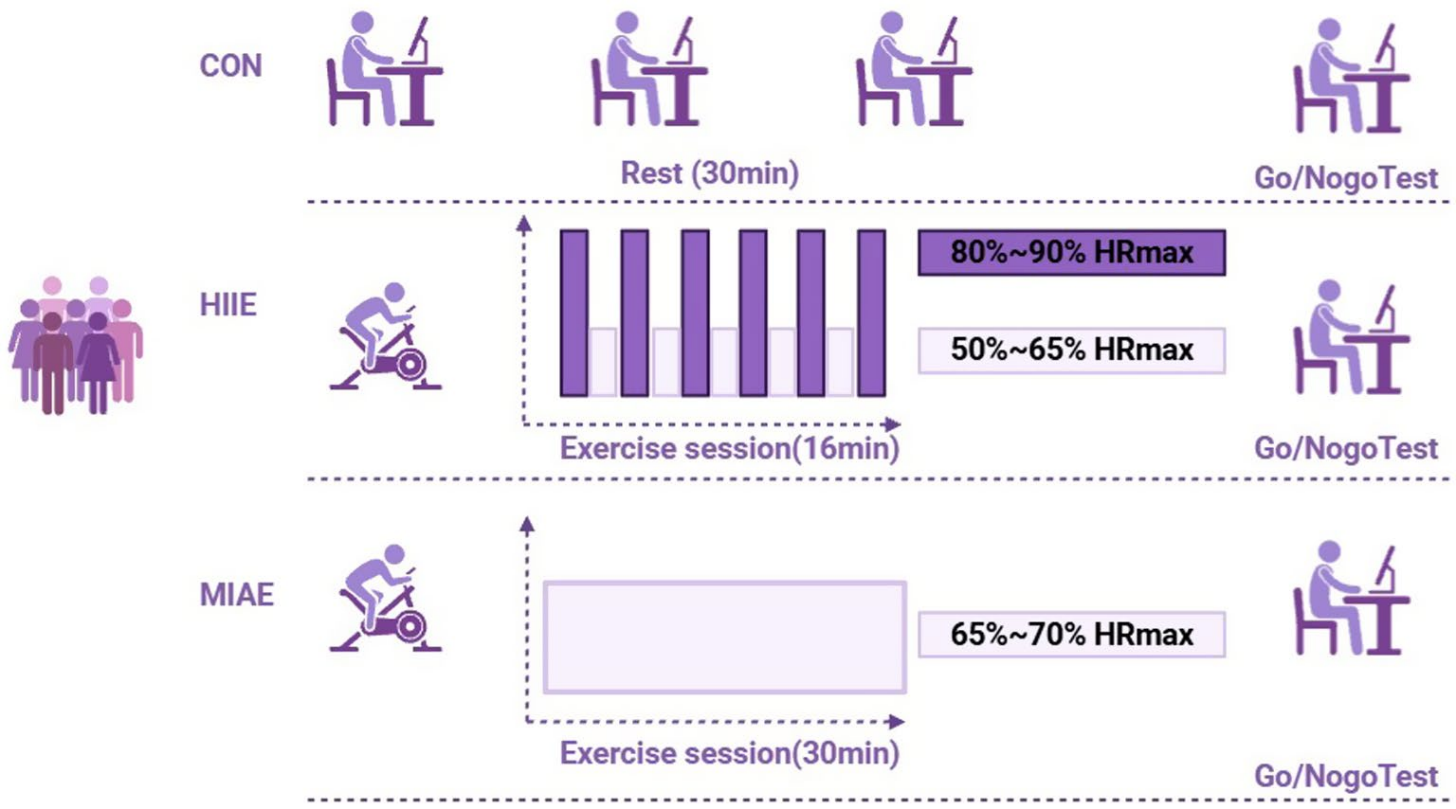
Exercise protocol.

Participants were instructed to maintain target heart rate zones throughout the sessions, with continuous heart rate monitoring via Polar monitors. Given the higher body weight of obese individuals, a stationary cycle ergometer was used as the exercise modality to ensure safety and minimize joint strain (Tilinca et al., 2016). As demonstrated in Figure 2.

### 2.6 Experimental procedure

Each participant completed three laboratory sessions: one resting control session and two exercise sessions (HIIE and MIAE). The order of the two exercise sessions was counterbalanced using a Latin square design to minimize order effects. Participants were randomly assigned to different session sequences. To reduce potential carry-over effects between conditions, a minimum wash-out period of 48 h was ensured between any two sessions, with an average interval of 3.1 ± 0.8 days. If scheduling conflicts arose, the next session was postponed to the same weekday of the following week to maintain consistency.

All sessions were scheduled at the same time of day and on the same day of the week across participants to control for circadian influences. Participants were instructed to sleep at least 7 h the night before, stay well hydrated, and avoid caloric intake for at least 4 h prior to testing. They were also asked to refrain from caffeine and vigorous exercise for 24 h before each session (Carbine et al., 2017).

Immediately following each exercise session, participants dried off sweat with a towel and blow-dried their scalp to avoid interference with EEG recordings. The EEG cap was applied after their heart rate returned to baseline. The food-related Go/NoGo task was then performed while EEG signals were recorded.

### 2.7 Data recording and analysis

#### 2.7.1 Behavioral data

For each participant, the mean reaction time and standard deviation were extracted from the food-related Go/NoGo task. Prior to analysis, outliers and extreme values were removed from each behavioral measure using the criterion of ±3 standard deviations from the individual mean. For the food-related Go/NoGo task, a three-way repeated measures ANOVA was conducted for each dependent variable (Go reaction time, Go accuracy, and NoGo accuracy), with the following factors: gender (male, female; between-subjects), exercise condition (resting control, HIIE, MIAE), image category (high-calorie, low-calorie, neutral).

#### 2.7.2 EEG data

EEG data were recorded using a 64-channel system (Brain Products, Germany) with a sampling rate of 1,000 Hz. FCz served as the online reference electrode and AFz as the ground. Scalp impedance was maintained below 5 kΩ for all electrodes. Electrooculographic (EOG) activity was monitored throughout the experiment, and TP9 and TP10 were used as offline reference electrodes during re-referencing. EEG preprocessing was conducted using MATLAB and EEGLAB. Raw signals were band-pass filtered at 0.1–30 Hz, and epochs were segmented from −200 ms to 1,000 ms relative to stimulus onset, with a 200 ms pre-stimulus baseline correction applied. Artifact rejection was performed by automatically excluding epochs with voltage fluctuations exceeding ±100 μV at any electrode, to eliminate artifacts caused by blinks, muscle activity, or movement. On average, 52 to 65 valid trials were retained per condition after artifact rejection, depending on the participant and condition. Based on previous literature on inhibitory control, two ERP components were analyzed: N2 (180–230 ms post-stimulus) and P3 (400–600 ms post-stimulus). Analyses focused on three electrode sites—Fz (frontal), Cz (central), and Pz (parietal)—representing key cortical regions involved in cognitive control. For ERP data (N2 and P3 mean amplitudes), a five-way repeated measures ANOVA was performed, with: Gender (male, female; between-subjects), exercise condition (resting control, HIIE, MIAE), trial type (Go, NoGo), image category (high-calorie, low-calorie, neutral), electrode site (Fz, Cz, Pz). Mauchly’s test was used to assess the sphericity assumption, and Greenhouse–Geisser corrections were applied when necessary.

For significant interaction effects, simple effects analyses were conducted, and Bonferroni correction was applied for multiple comparisons (adjusted *α* = 0.05/*n*). Effect sizes were reported using partial eta-squared (*η*^2^_p_) to indicate the magnitude of observed effects.

## 3 Results

### 3.1 Behavioral results

#### 3.1.1 Reaction time

A 3 (exercise condition) × 3 (image category) repeated measures ANOVA was performed for each gender group. Significant main effects of exercise condition were found for both females and males: Females: *F*(2,12) = 5.06, *p* < 0.05, *η*^2^_p_ = 0.46; males: *F*(2,12) = 11.69, *p* < 0.01, *η*^2^_p_ = 0.66; *Post hoc* comparisons showed that reaction times under both HIIE and MIAE were significantly faster than in the resting control condition (*p*s < 0.05). Notably, male participants exhibited a trend toward shorter reaction times under HIIE compared to MIAE (*p* = 0.058).

**Figure 3:**
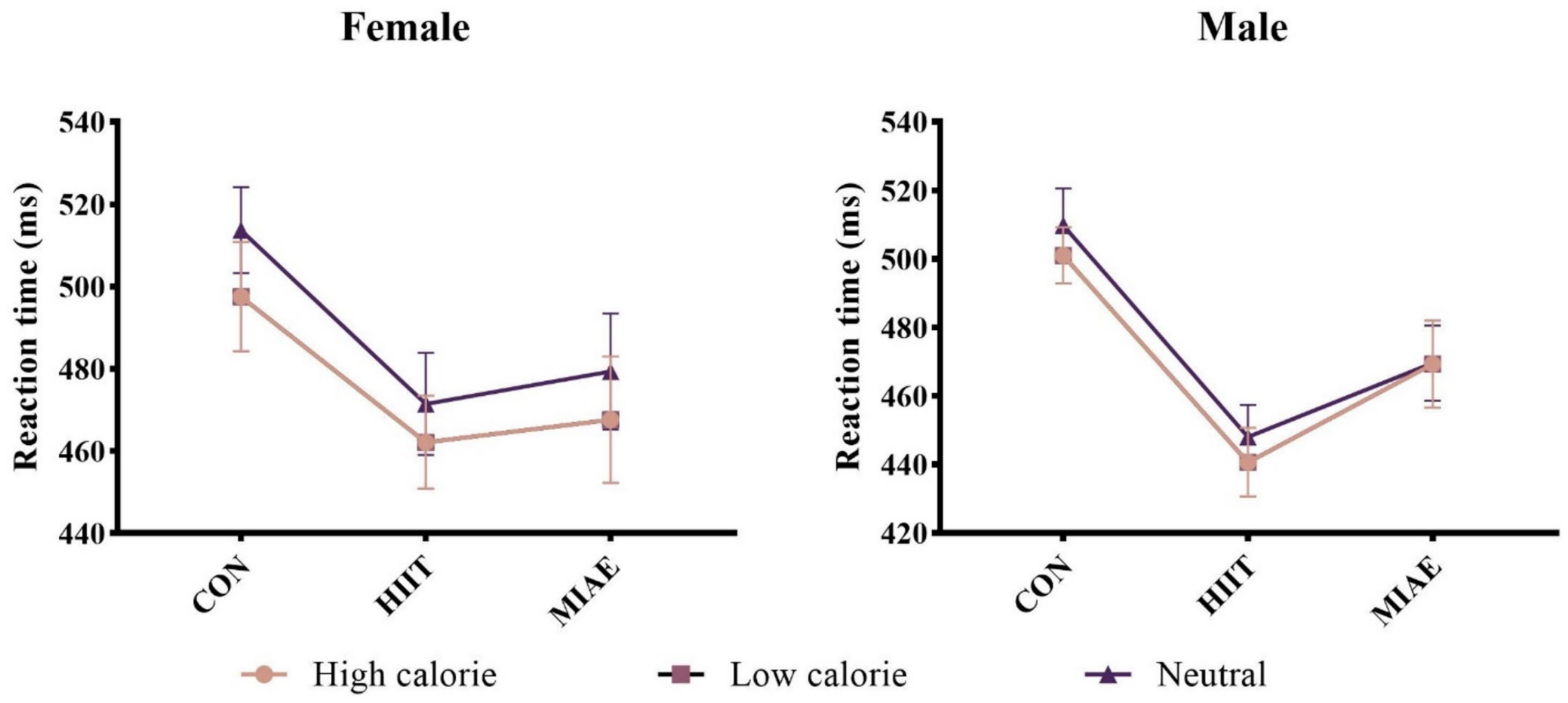
Food Go/NoGo task response time under different conditions.

Female group: 504.47 ± 10.81 ms (rest) vs. 462.27 ± 10.71 ms (HIIE) vs. 469.89 ± 13.10 ms (MIAE). Male group: 501.36 ± 9.92 ms (rest) vs. 441.13 ± 8.57 ms (HIIE) vs. 463.18 ± 10.70 ms (MIAE). In addition, both groups showed a significant main effect of image category: Females: *F*(2,12) = 4.75, *p* < 0.05, *η*^2^_p_ = 0.44; Males: *F*(2,12) = 7.79, *p* < 0.01, *η*^2^_p_ = 0.57. Participants responded faster to low-calorie food images compared to neutral images (*p*s < 0.05). In males, reaction times to high-calorie images were significantly slower than those to low-calorie images (*p* < 0.05). Females: 475.77 ± 10.46 ms (high-calorie) vs. 472.72 ± 8.78 ms (low-calorie) vs. 488.14 ± 10.52 ms (neutral); males: 470.34 ± 7.47 ms (high-calorie) vs. 459.53 ± 8.18 ms (low-calorie) vs. 475.81 ± 8.55 ms (neutral); no significant interaction effects were observed (*p*s > 0.05). As demonstrated in Figure 3.

#### 3.1.2 Accuracy

**Figure 4:**
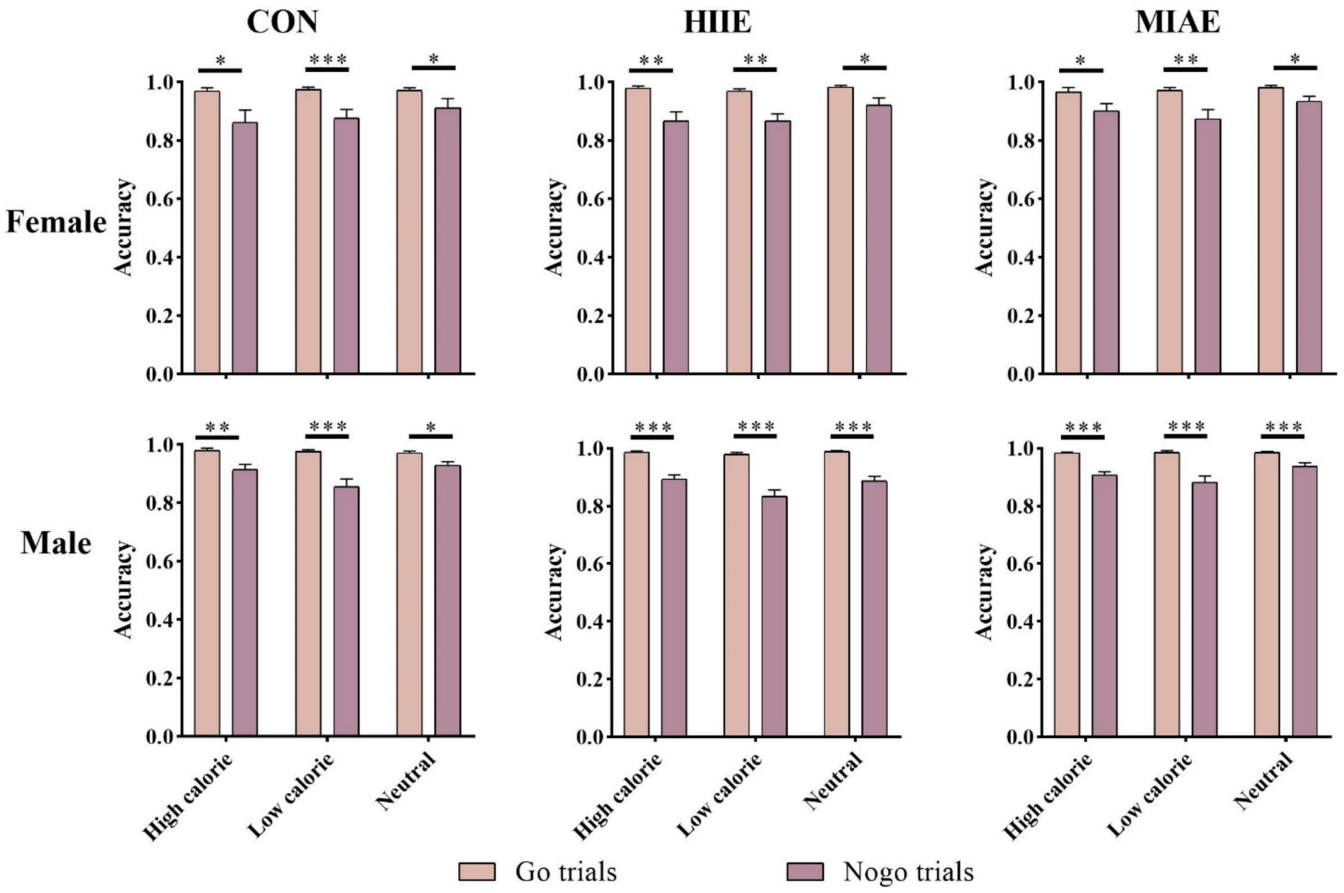
Food Go/NoGo task correctness under different conditions.

A repeated measures ANOVA revealed significant main effects of image category and trial type for both female and male participants: Females: *F*(1,13) = 13.02, *p* < 0.01, *η*^2^_p_ = 0.50; males: *F*(1,13) = 89.33, *p* < 0.001, *η*^2^_p_ = 0.87; across all exercise conditions, participants demonstrated significantly higher accuracy in Go trials compared to NoGo trials (*p*s < 0.01). Furthermore, the accuracy gap between Go and NoGo trials was greater for low-calorie images than for high-calorie or neutral images in both groups. For female participants, the difference in accuracy between Go and NoGo trials was less pronounced between high- and low-calorie conditions: resting condition: 0.97 ± 0.01 (Go) vs. 0.88 ± 0.03 (NoGo); HIIE: 0.97 ± 0.01 (Go) vs. 0.87 ± 0.02 (NoGo); MIAE: 0.98 ± 0.01 (Go) vs. 0.92 ± 0.02 (NoGo); in contrast, male participants exhibited a larger Go-NoGo accuracy gap under high-calorie image conditions: resting condition: 0.98 ± 0.004 (Go) vs. 0.90 ± 0.01 (NoGo); HIIE: 0.98 ± 0.004 (Go) vs. 0.86 ± 0.02 (NoGo); MIAE: 0.98 ± 0.003 (Go) vs. 0.92 ± 0.01 (NoGo); no other significant interactions were observed (*p*s > 0.05). As demonstrated in Figure 4.

### 3.2 EEG results

#### 3.2.1 N2 amplitude

**Figure 5:**
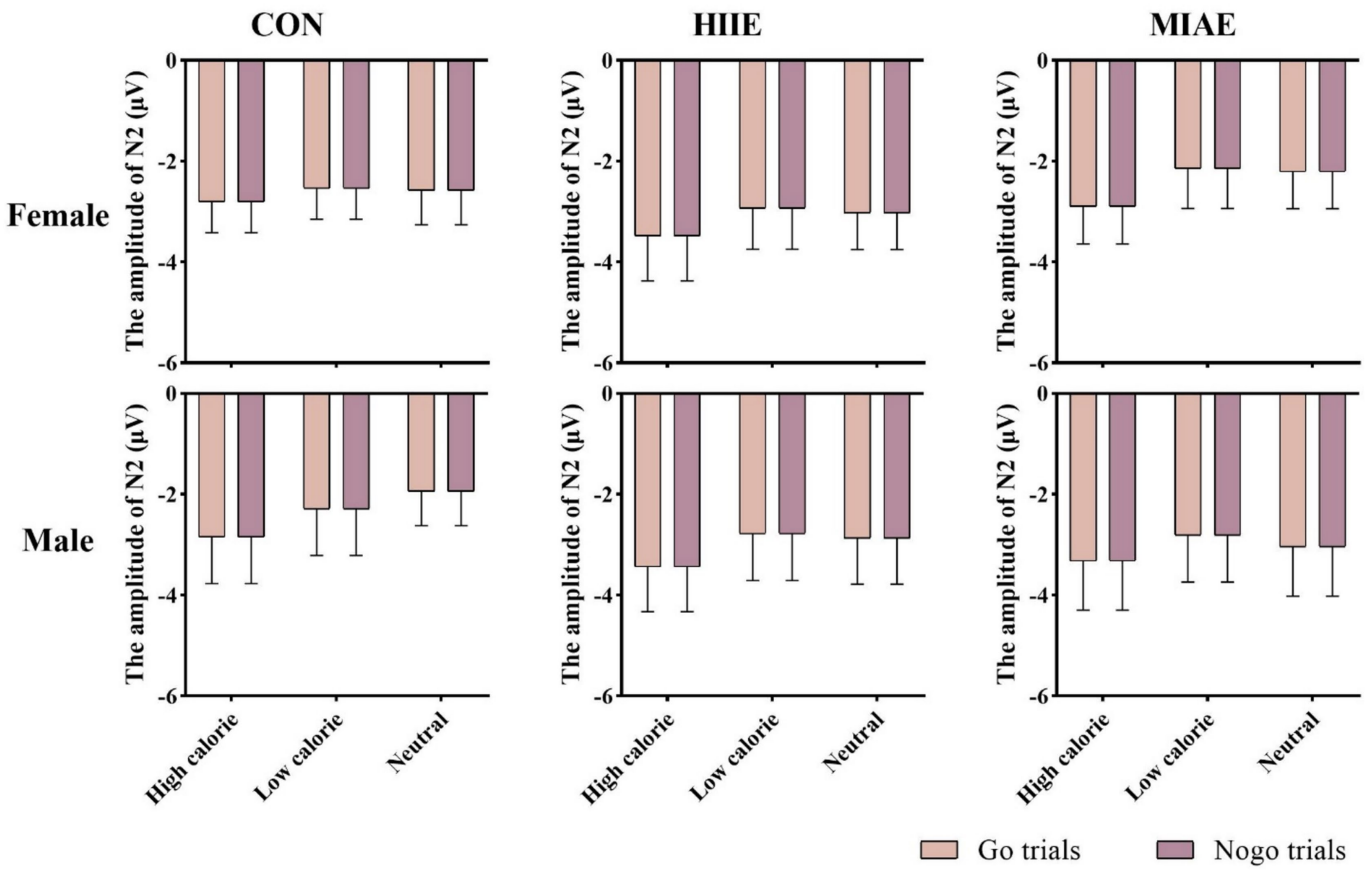
N2 amplitude of the food Go/Nogo task under different conditions at the Fz point.

**Figure 6:**
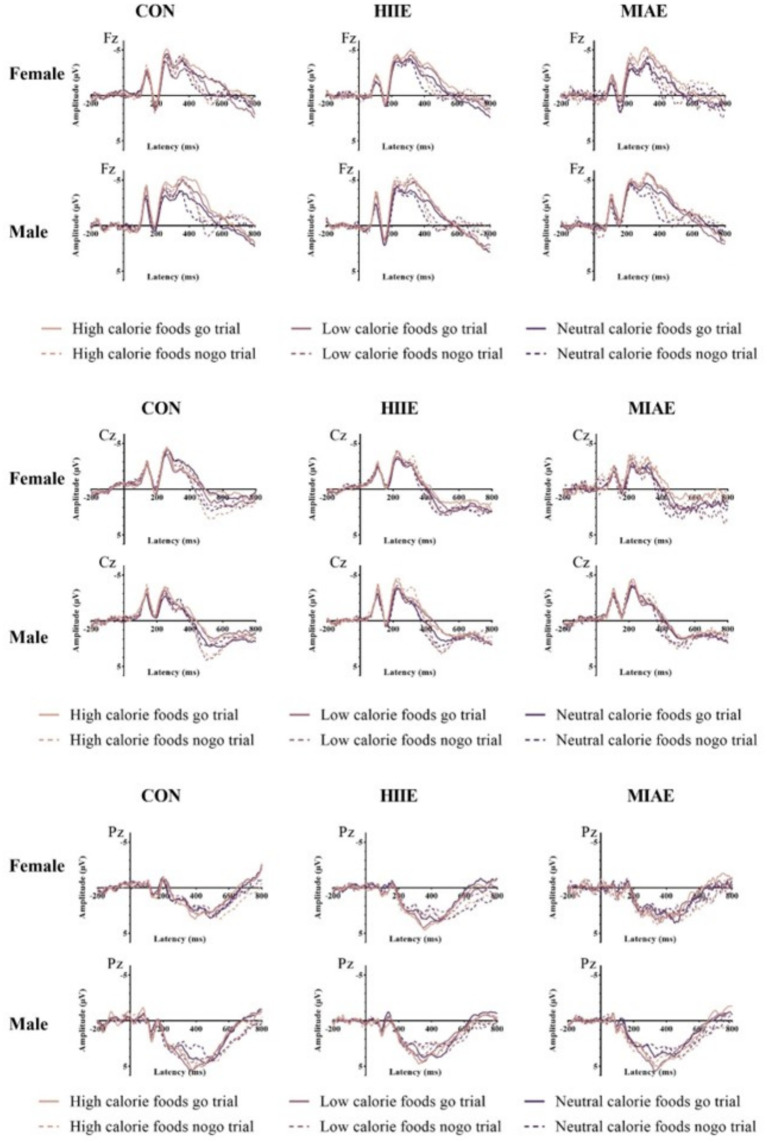
Waveforms of food Go/Nogo task N2, P3 amplitudes under different motion conditions at Fz/Cz/Pz points.

**Figure 7:**
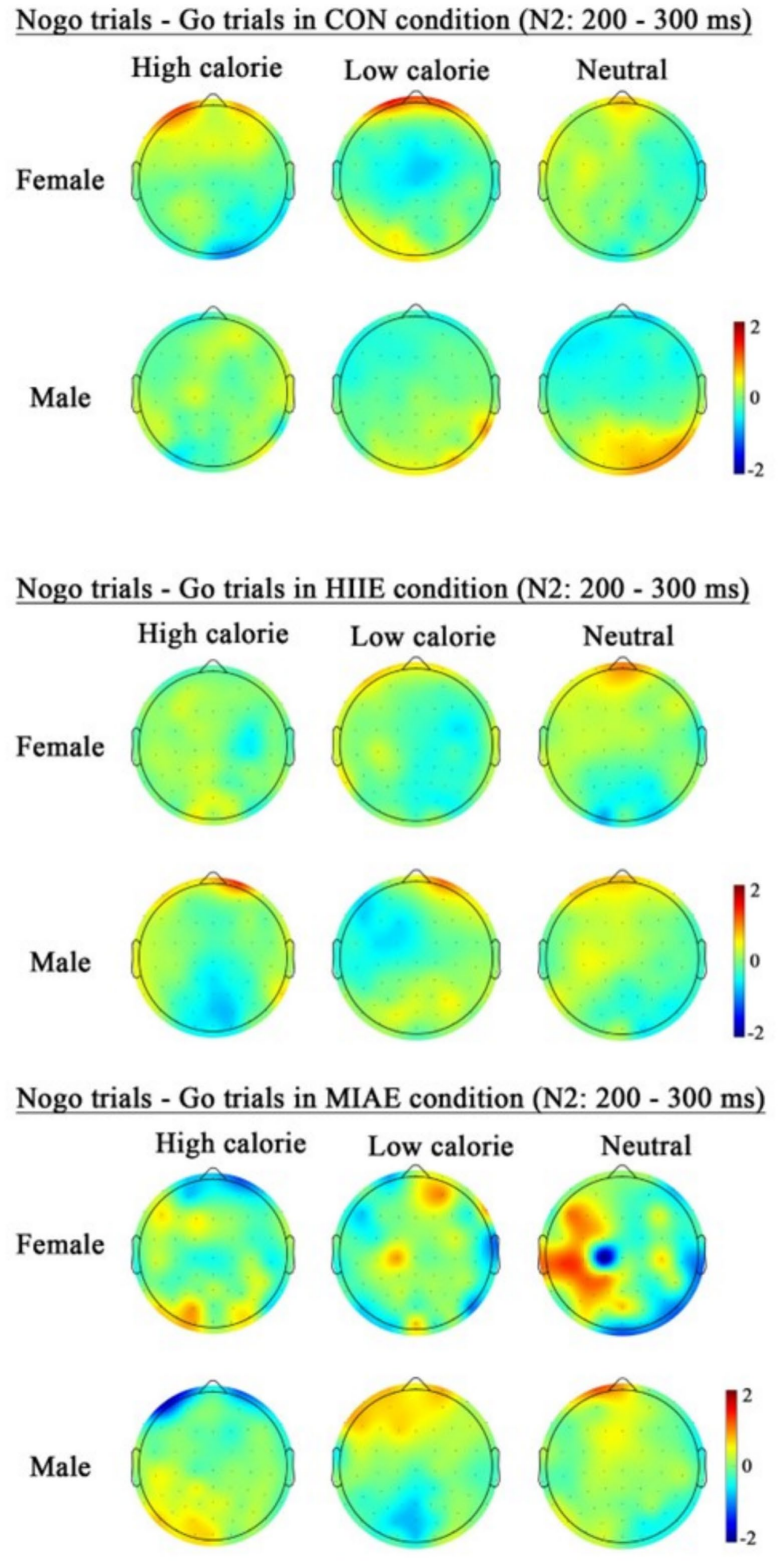
Topography of the N2 amplitude of the FoodGo/Nogo mission under different conditions.

At Fz site a significant main effect of image category was observed at the Fz electrode for both females and males: females: *F*(2,12) = 21.94, *p* < 0.001, *η*^2^_p_ = 0.79; males: *F*(2,12) = 13.59, *p* < 0.001, *η*^2^_p_ = 0.69. *Post hoc* analyses revealed: In females, high-calorie images elicited significantly larger N2 amplitudes compared to both low-calorie and neutral images (*ps* < 0.001), while the difference between low-calorie and neutral images was not significant (*p* > 0.05). High-calorie: −3.81 ± 0.51 μV; low-calorie: −3.14 ± 0.49 μV; neutral: −2.96 ± 0.41 Μv. In males, high-calorie images also elicited greater N2 amplitudes than both low-calorie and neutral images (*p*s < 0.01), and low-calorie images showed significantly larger amplitudes than neutral images (*p* < 0.001): high-calorie: −4.35 ± 0.43 μV; low-calorie: −3.85 ± 0.43 μV; neutral: −3.36 ± 0.42 μV; to further explore group differences—despite no significant three-way interaction—a simple effects analysis was performed: in females, under the HIIE condition and in response to low-calorie food images, NoGo trials elicited significantly larger N2 amplitudes than Go trials (*p* < 0.02): Go: −2.34 ± 0.57 μV; NoGo: −2.93 ± 0.66 μV. No such differences were found under other exercise or image conditions, nor in male participants (*p*s > 0.05). As demonstrated in Figures 5–7.

**Figure 8:**
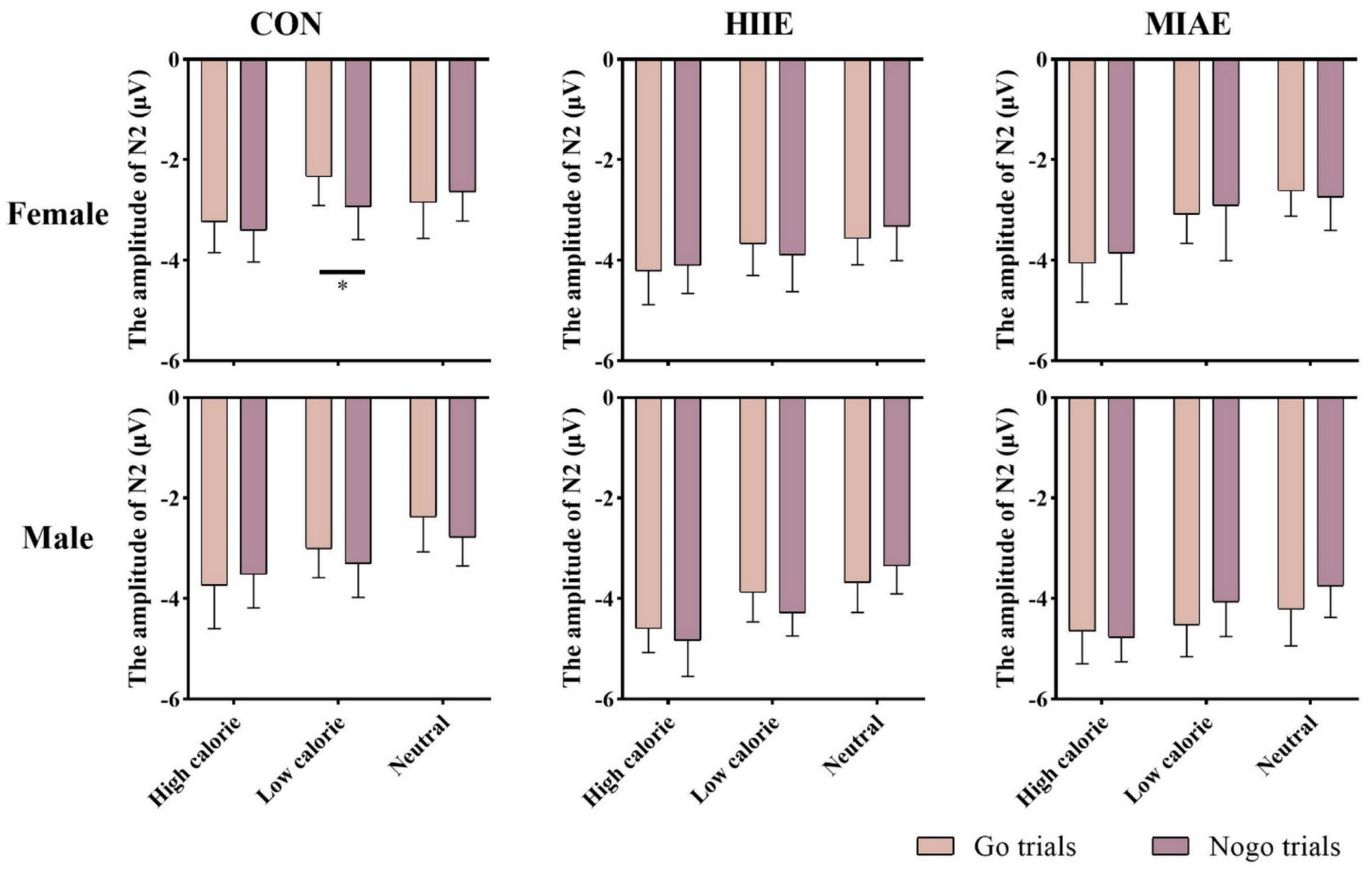
N2 amplitude of the food Go/Nogo task under different conditions at the Cz point.

At the Cz electrode, a significant main effect of image category was observed in both females and males: Females: *F*(2,12) = 4.59, *p* < 0.05, *η*^2^_p_ = 0.43; males: *F*(2,12) = 9.35, *p* < 0.01, *η*^2^_p_ = 0.61. *Post hoc* analysis showed: For both groups, high-calorie food images elicited significantly larger N2 amplitudes compared to both low-calorie and neutral images (*p*s < 0.05). No significant difference was found between low-calorie and neutral images (*p*s > 0.05). Females: high-calorie: −3.15 ± 0.63 μV; low-calorie: −2.62 ± 0.66 μV; neutral: −2.62 ± 0.62 μV; males: high-calorie: −3.29 ± 0.79 μV; low-calorie: −2.71 ± 0.82 μV; neutral: −2.61 ± 0.73 μV. Further simple effects analyses revealed no significant differences between Go and NoGo trials under any condition in either group (*p*s > 0.05). As demonstrated in Figures 6–8.

**Figure 9:**
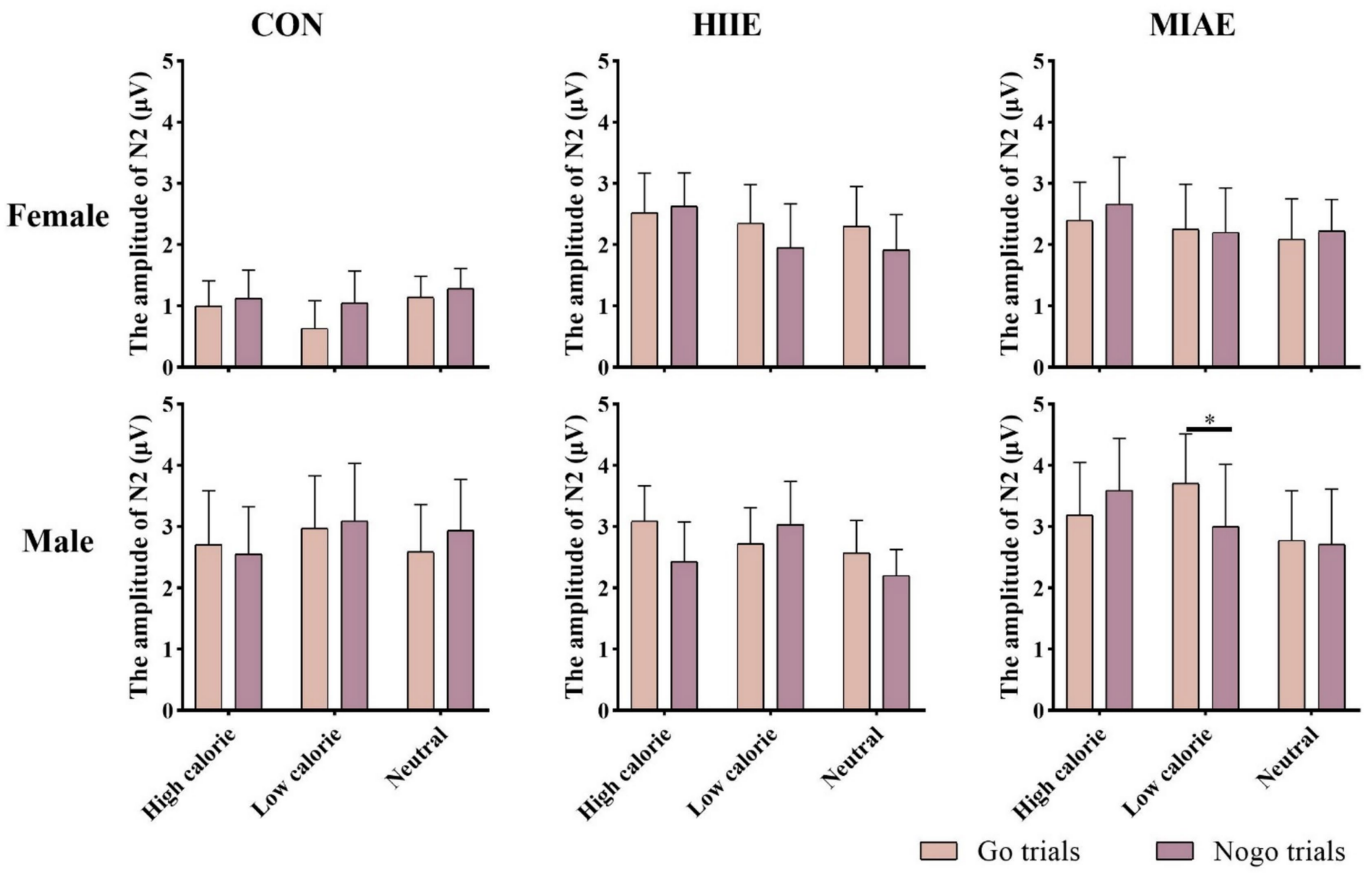
N2 amplitude of the food Go/Nogo task under different conditions at the Pz point.

At the Pz electrode, a marginally significant main effect of image category was found in the female group: Females: *F*(2,12) = 3.29, *p* = 0.073, *η*^2^_p_ = 0.35. *Post hoc* comparisons showed that high-calorie food images tended to elicit larger N2 amplitudes than low-calorie images (*p* = 0.061), while no significant differences were observed between either high- or low-calorie food and neutral images (*p*s > 0.05). High-calorie: −2.06 ± 0.38 μV; Low-calorie: −1.74 ± 0.42 μV; neutral: −1.83 ± 0.33 μV. In the male group, no significant main effect of image category was found (*p*s > 0.05). However, a significant three-way interaction between exercise condition, image category, and trial type was observed: males: *F*(2,10) = 4.84, *p* < 0.05, *η*^2^_p_ = 0.66. Further simple effects analysis indicated that, after MIAE, Go trials with low-calorie images elicited significantly more positive N2 amplitudes than NoGo trials: Go: 3.71 ± 0.81 μV; NoGo: 3.00 ± 0.90 μV. No significant Go–NoGo differences were observed under other exercise or image conditions (*p*s > 0.05). No significant effects were found for females at the Pz site under any condition (*p*s > 0.05). As demonstrated in Figures 6, 7, 9.

#### 3.2.2 P3 amplitude

**Figure 10:**
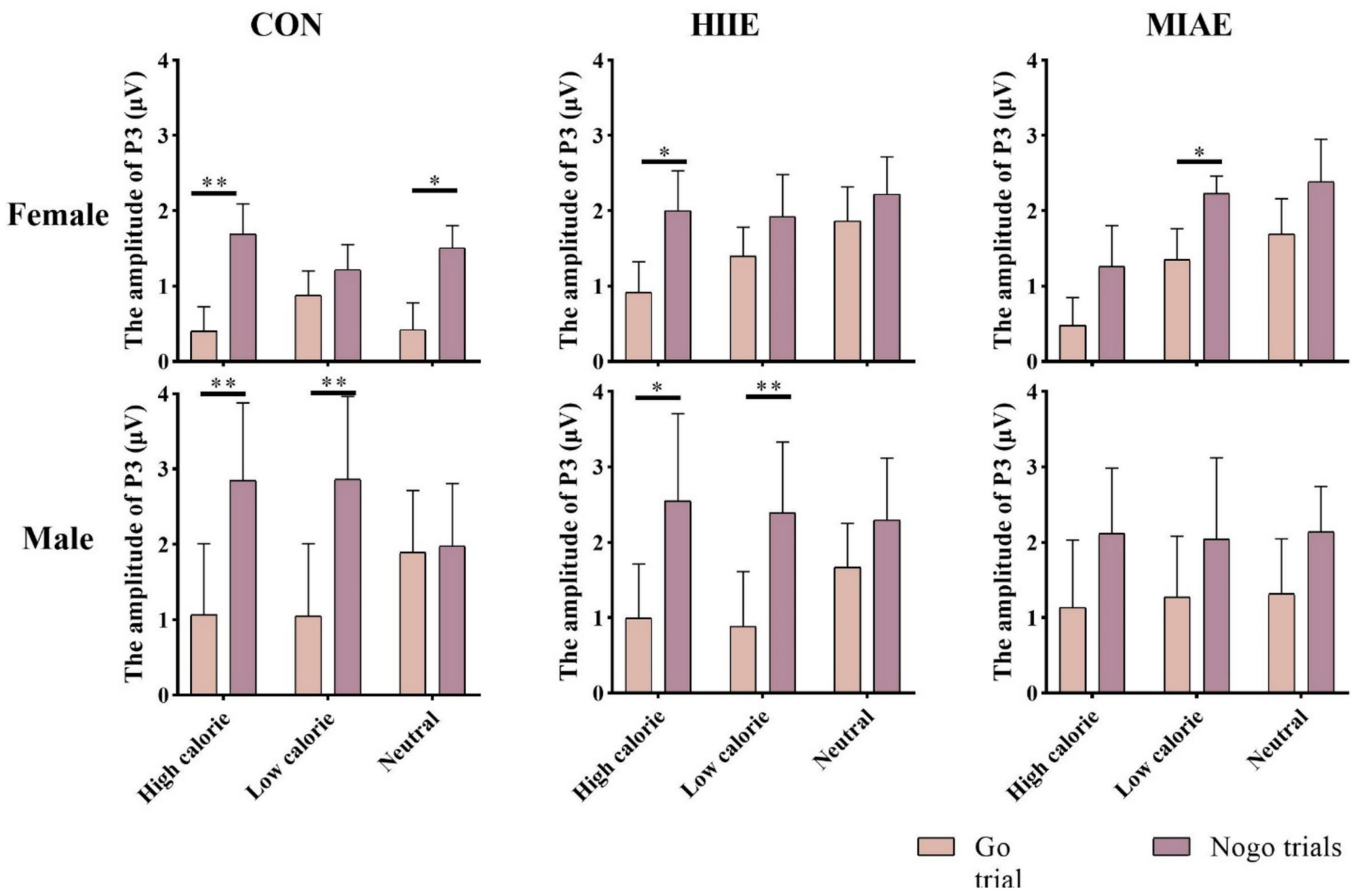
Food Go/NoGo task P3 amplitude for different conditions at point Fz.

**Figure 11:**
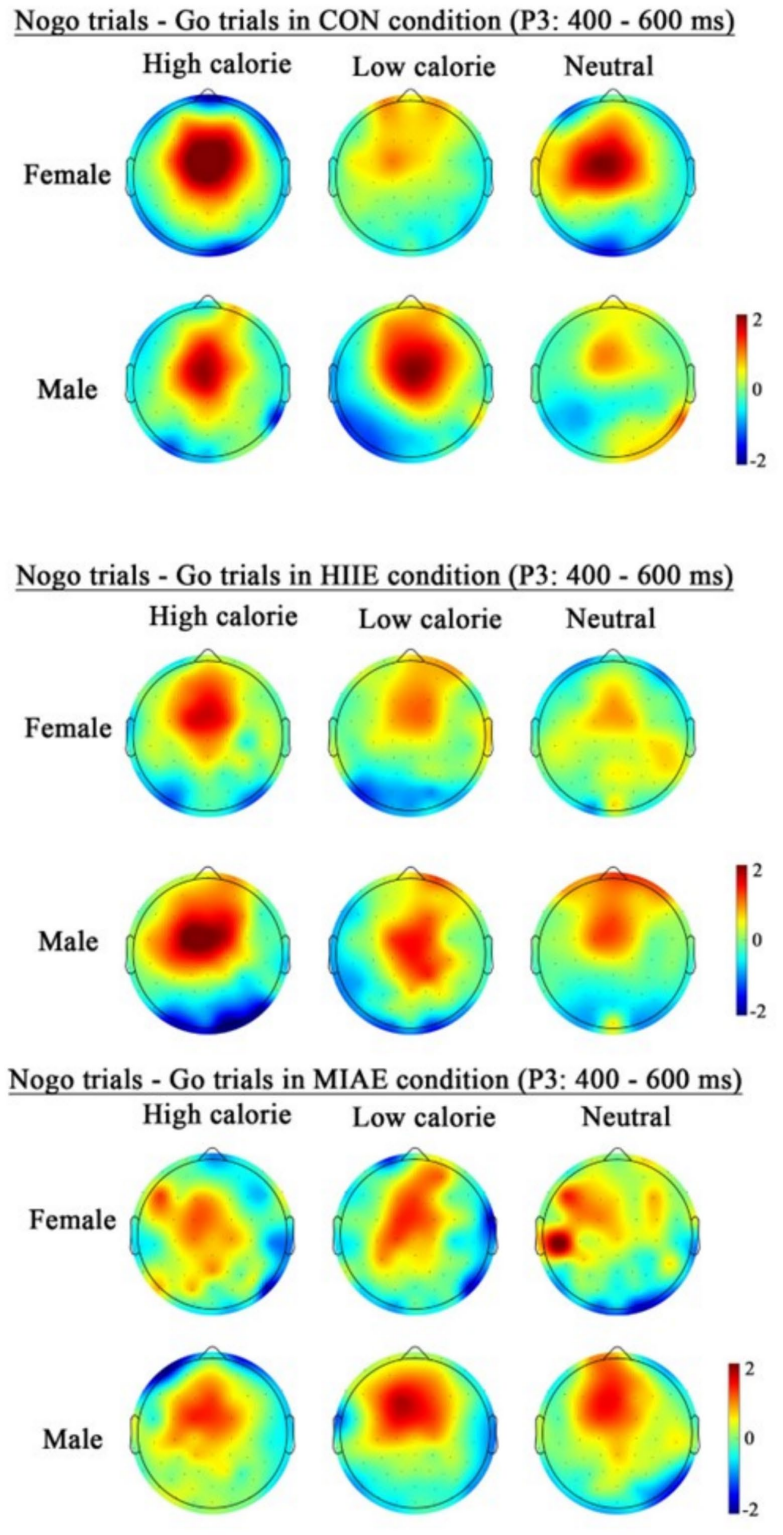
Topography of the P3 amplitude of the FoodGo/Nogo mission under different conditions.

At the Fz electrode, a significant main effect of image category was observed in the female group: Females: *F*(2,12) = 12.66, *p* = 0.001, *η*^2^_p_ = 0.69. *Post hoc* tests showed that high-calorie food images elicited more negative P3 amplitudes than both low-calorie and neutral images (*p*s < 0.05), with no significant difference between the latter two (*p* > 0.05). High-calorie: −1.33 ± 0.36 μV; low-calorie: −0.80 ± 0.29 μV; neutral: −0.67 ± 0.28 μV. In contrast, no significant main effect of image category was observed in males (*p*s > 0.05): high-calorie: −1.66 ± 0.57 μV; low-calorie: −1.35 ± 0.54 μV; neutral: −1.05 ± 0.44 μV; both females and males showed a significant main effect of trial type: females: *F*(1,13) = 25.06, *p* < 0.001, *η*^2^_p_ = 0.66; males: *F*(1,13) = 51.97, *p* < 0.001, *η*^2^_p_ = 0.80. In both groups, NoGo trials elicited significantly larger P3 amplitudes than Go trials: females: NoGo = −1.51 ± 0.31 μV vs. Go = −0.36 ± 0.33 μV; males: NoGo = −2.04 ± 0.52 μV vs. Go = −0.66 ± 0.51 μV. Further simple effects analysis showed that for females, the NoGo vs. Go P3 difference was significant in: All image types under resting and HIIE conditions; low-calorie images under MIAE. However, no significant difference was observed between NoGo and Go trials for high-calorie and neutral images under MIAE (*p*s > 0.05). In contrast, male participants showed consistent and significant NoGo–Go P3 differences under all conditions and image types (*p*s < 0.05). As demonstrated in Figures 6, 10, 11.

**Figure 12:**
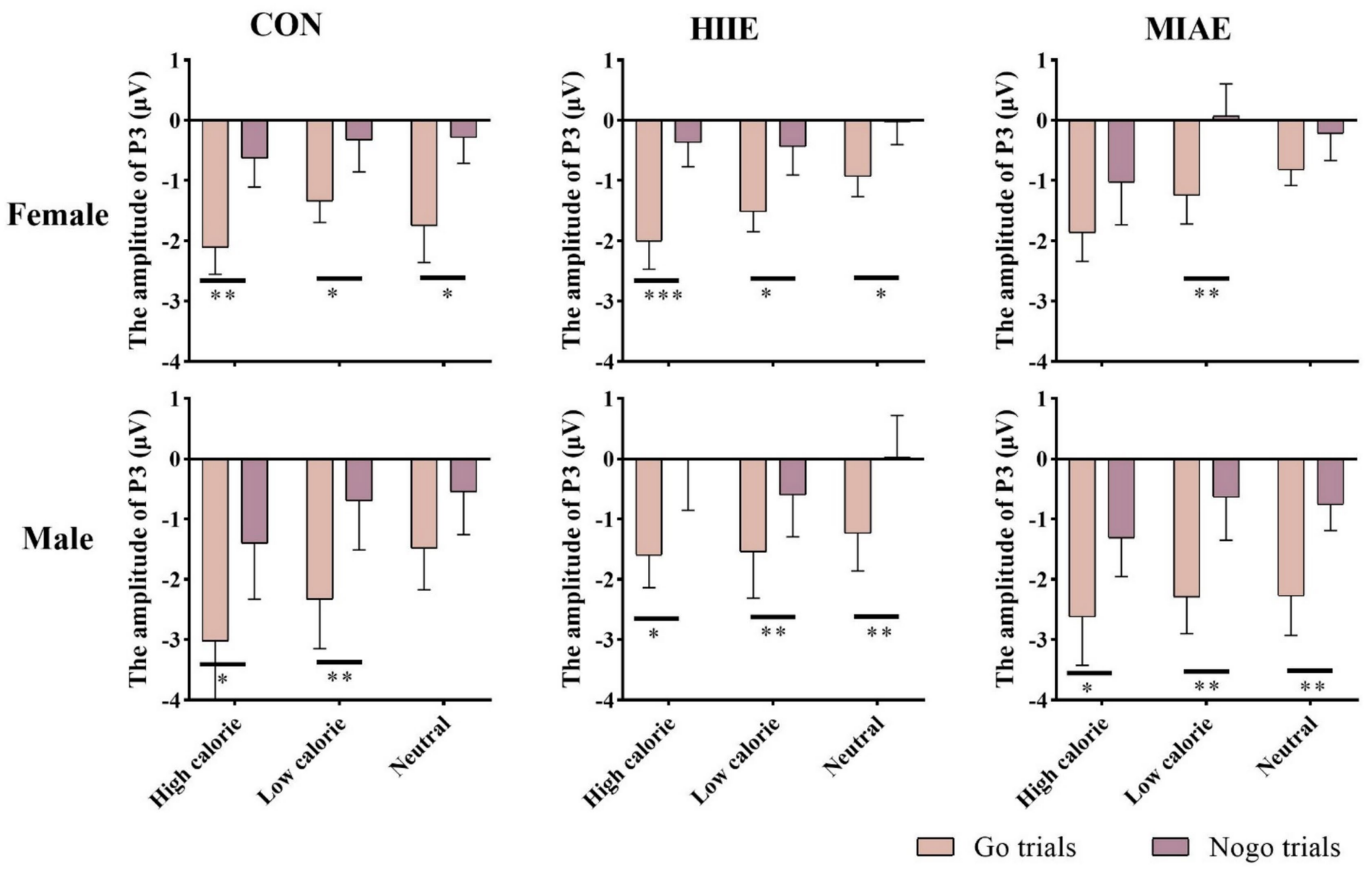
Food Go/NoGo task P3 amplitude for different conditions at point Cz.

At the Cz electrode, a significant main effect of image category was found in females: Females: *F*(2,12) = 4.03, *p* = 0.046, *η*^2^_p_ = 0.40. *Post hoc* comparisons indicated that neutral images elicited significantly higher P3 amplitudes than high-calorie images (*p* < 0.05), while other pairwise differences were not significant (*p*s > 0.05): high-calorie: 1.13 ± 0.26 μV; low-calorie: 1.50 ± 0.20 μV; neutral: 1.68 ± 0.22 μV. No main effect of image category was observed in males (*p*s > 0.05): high-calorie: 1.79 ± 0.78 μV; low-calorie: 1.75 ± 0.77 μV; neutral: 1.88 ± 0.60 μV. Both groups showed significant main effects of trial type: females: *F*(1,13) = 13.83, *p* < 0.01, *η*^2^_p_ = 0.52; Males: *F*(1,13) = 13.05, *p* < 0.01, *η*^2^_p_ = 0.50. NoGo trials elicited higher P3 amplitudes than Go trials: females: NoGo = 1.83 ± 0.27 μV vs. Go = 1.05 ± 0.20 μV; Males: NoGo = 2.36 ± 0.81 μV vs. Go = 1.25 ± 0.64 μV. Further analysis showed that: in females, significant NoGo–Go differences appeared under: rest condition with high-calorie and neutral images; HIIE with high-calorie images; MIAE with low-calorie images. In males, the NoGo–Go difference was significant under: rest and HIIE for high-calorie and neutral images. As demonstrated in Figures 6, 11, 12.

**Figure 13:**
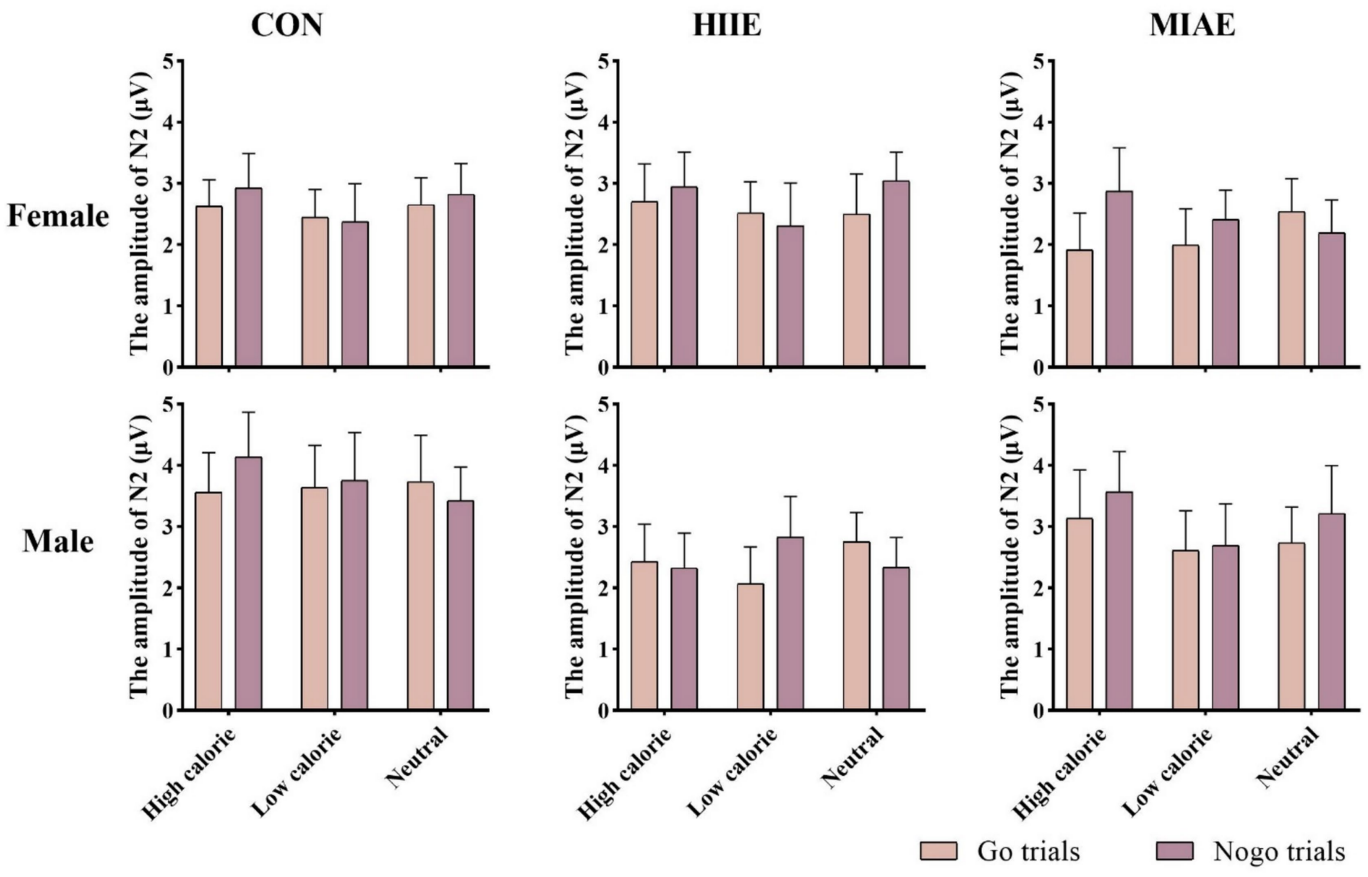
Food Go/NoGo task P3 amplitude for different conditions at point Pz.

At the Pz electrode, a significant main effect of image category was found in the female group: Females: *F*(2,12) = 9.46, *p* < 0.01, *η*^2^_p_ = 0.61. *Post hoc* analysis showed that high-calorie images elicited significantly greater P3 amplitudes than low-calorie images (*p* < 0.01), while neither high- nor low-calorie images differed significantly from neutral images (*p*s > 0.05): high-calorie: 2.66 ± 0.42 μV; low-calorie: 2.35 ± 0.42 μV; neutral: 2.63 ± 0.38 μV. No significant main effects or interactions were found in the male group at Pz (*p*s > 0.05). To further explore differences between Go and NoGo trials, a simple effects analysis was conducted for each image type under each exercise condition. However, no significant Go–NoGo P3 differences were observed at the Pz site in either males or females under any condition (*p*s > 0.05). As demonstrated in Figures 6, 11, 13.

## 4 Discussion

In terms of behavioral performance related to food-specific inhibitory control, both short-term high-intensity interval exercise (HIIE) and moderate-intensity aerobic exercise (MIAE) facilitated faster reaction times in obese adults. Notably, HIIE exerted a greater enhancement in response speed among obese males compared to MIAE. However, ERP data revealed no significant modulation in N2 or P3 amplitudes in this subgroup following HIIE. This dissociation between behavioral performance and ERP outcomes precludes strong mechanistic conclusions, and we thus interpret these findings with caution. One plausible explanation, based on previous neurophysiological literature, is that HIIE may induce non-inhibitory arousal- or motivation-related changes in neural functioning, potentially involving the prefrontal–striatal circuitry. Supporting this, earlier studies have reported that HIIE acutely elevates catecholamine levels (e.g., norepinephrine) and brain-derived neurotrophic factor (BDNF) in obese individuals (Athanasiou et al., 2023; Ceylan et al., 2023), which may influence motor-associative circuits or reward–motivation pathways in the ventral striatum (Gökçe et al., 2024). While these effects may contribute to faster behavioral responses, further investigation using targeted neurophysiological markers is necessary to clarify the underlying mechanisms (Gökçe et al., 2024). Such pathways can accelerate behavioral output without the recruitment of additional inhibitory resources. These findings align with the neural efficiency hypothesis, which posits that acute metabolic stress induced by HIIE activates the sympathetic–adrenal medullary axis, elevates arousal, and enhances motor cortex excitability, enabling faster responses with less cortical effort (Inoue et al., 2020). Given the regional specificity of N2 and P3 components across cognitive processing stages, with N2 at Fz typically reflecting conflict detection and P3 at Pz associated with higher-order cognitive processes such as attentional allocation, this study focused on examining amplitude changes at these critical sites. While inhibitory control is recognized as a product of distributed neural coordination, the regulatory role of individual regions—especially the frontal cortex—remains fundamental (Paret et al., 2019). The modified food-related Go/NoGo task used in this study incorporated high-calorie, low-calorie, and neutral food images to simulate real-life dietary decision-making contexts, thereby enabling more ecologically valid assessments of food-specific inhibition (Bartholdy et al., 2016). Results showed that high-calorie food images elicited significantly larger N2 amplitudes than both low-calorie and neutral stimuli in both male and female obese participants, reflecting increased conflict monitoring demands evoked by energy-dense cues (Hoyniak and Petersen, 2019). Additionally, NoGo trials elicited greater P3 amplitudes than Go trials, indicating that response inhibition required greater cognitive resources than execution, further supporting the validity of the Go/NoGo paradigm used in this context (Wang et al., 2022).

Given the limited number of studies and the inconsistent findings regarding the acute effects of exercise on food-related inhibitory control, further research using event-related potential (ERP) techniques is warranted. These investigations should aim to determine whether short-term exercise enhances food inhibition by strengthening inhibitory processes toward high-calorie, palatable foods, or by increasing motivational salience toward healthier, low-calorie food choices, thereby indirectly reducing high-calorie food intake (Lowe et al., 2016, 2019). Wang reported that, in males, acute exercise reduced activation in the orbitofrontal cortex (OFC) during food inhibition tasks, which was associated with lower self-reported hunger. This suggests a perceptual role of the OFC in food-related inhibitory processes, a pattern not observed in females, thereby indicating potential sex-specific modulation in food inhibition following exercise interventions. In the current study, under the MIAE condition, obese males exhibited no significant difference in N2 amplitude between Go and NoGo trials for low-calorie food images. Typically, the N2 component reflects conflict monitoring mediated by the anterior cingulate cortex (ACC) or early response inhibition via the prefrontal-parietal network (Folstein and Van Petten, 2008). In classical Go/NoGo paradigms, NoGo trials usually elicit larger N2 amplitudes due to the need to suppress a dominant response—an index of “response–non-response” conflict detection (Folstein and Van Petten, 2008). However, in this study, the Go trials with low-calorie images evoked greater N2 amplitudes than NoGo trials, which may reflect a task-specific redistribution of cognitive resources. When low-calorie images serve as Go stimuli and high-calorie images as NoGo stimuli, obese males—possibly due to prolonged exposure to high-calorie environments—may display an implicit approach bias, or an automatic response tendency toward such cues. MIAE, by enhancing cognitive control via the prefrontal–striatal pathway (Guiney et al., 2015), may prompt participants to allocate greater attentional resources during Go trials to overcome habitual neglect of low-calorie cues, which are typically less motivationally salient. Thus, the elevated N2 amplitude in Go trials may not reflect classical inhibition demands, but rather enhanced ACC-based conflict monitoring related to the initiation of a non-dominant response (i.e., approaching low-calorie food). Furthermore, MIAE may improve prefrontal oxygenation (Tsukamoto et al., 2016) and strengthen dorsolateral prefrontal–parietal connectivity (Legget et al., 2016), facilitating early attentional capture of low-calorie stimuli. The increased N2 in Go trials may also represent more refined perceptual processing—such as the recognition of health-related attributes—requiring ACC-DLPFC cooperation to suppress the automatic attentional bias toward high-calorie cues (Hare et al., 2009). In this context, the increased N2 amplitude reflects a strategic cognitive shift in obese males post-exercise—from passive neglect to active recognition of low-calorie food cues.

In the P3 amplitude analysis at the Fz electrode, female participants with obesity showed no significant difference between NoGo and Go trials under the MIAE condition when responding to high-calorie food images. Typically, NoGo trials elicit larger P3 amplitudes than Go trials, as they represent greater inhibitory demands. This is especially true for high-calorie stimuli, which are known to require elevated cognitive control due to their high reward salience. However, the absence of a significant NoGo–Go P3 difference in this study suggests that, under MIAE, obese females no longer recruited additional inhibitory resources when exposed to high-calorie cues. Previous research has found that acute MIAE attenuates neural responses to food-related stimuli, particularly compared to non-food stimuli, indicating that exercise may play a positive role in reducing attentional allocation to food cues. The present findings extend this perspective, suggesting that MIAE not only modulates attention to food cues, but may also enhance inhibitory responses to high-calorie foods. Moreover, the observed increase in N2 amplitude to low-calorie Go trials among obese females after MIAE implies enhanced conflict monitoring, likely mediated by the anterior cingulate cortex (ACC). This supports the hypothesis that MIAE improves prefrontal oxygenation and insulin sensitivity, which may optimize early ACC-based categorization of food stimuli (e.g., distinguishing low- vs. high-calorie foods). The disappearance of the NoGo–Go P3 difference in high-calorie conditions also indicates a reorganization of late-stage inhibitory resource allocation. This could result from: increased serotonergic transmission via upregulation of tryptophan hydroxylase activity, reducing the reward salience of high-calorie foods (Strasser et al., 2016). Enhanced hippocampal–prefrontal synaptic plasticity, promoting adaptive cognitive strategies such as implicit attentional avoidance, which reduce reliance on late-stage P3-linked cognitive effort (Crabtree et al., 2014). Additionally, MIAE may suppress excessive activation of striatal dopamine D2 receptors, thereby dampening the anticipated reward conflict associated with high-calorie cues. This mechanism could weaken the positive feedback loop in NoGo tasks that typically links inhibitory demand to resource allocation (Robison et al., 2018). In sum, the absence of a NoGo–Go P3 difference for high-calorie stimuli under MIAE may reflect a shift from active suppression to automatic processing in obese females. Although this interpretation is consistent with the neural efficiency hypothesis and previous literature, we acknowledge that it remains a speculative conclusion within the scope of the current data. The ERP results did not provide direct neural markers to validate a shift in inhibitory strategy, such as from active suppression to automatic processing. Therefore, this mechanism should still be regarded as a theoretical hypothesis that requires further validation. Future studies may incorporate additional neurophysiological tools, such as source localization or connectivity analyses, to further clarify the specific neural mechanisms underlying these ERP patterns. This supports the notion that MIAE can induce a neural efficiency reorganization of food-specific inhibitory control, rendering high-calorie stimuli less cognitively prioritized during evaluation, thereby balancing neural responses in the food-related Go/NoGo task.

It should be noted that ERP data were only recorded following each experimental condition (HIIE, MIAE, and resting control), and no pre-exercise baseline was collected. This decision was primarily based on concerns regarding participant fatigue and the potential for practice effects that may confound the comparability of ERP responses across sessions. However, the absence of a pre-intervention baseline introduces limitations in attributing observed ERP differences solely to exercise-induced changes, as session-to-session variability cannot be completely ruled out. Secondly, the initial sample size estimation was conducted using G*Power based on a typical three-condition within-subject design model, without incorporating sex as a predefined factor. As a result, the current sample size (16 participants per sex) may be underpowered to detect sex × condition interaction effects, and related findings should be interpreted as exploratory. Thirdly, although the sample size (n = 32) was statistically justified, the study population consisted exclusively of young obese adults from China, which limits the generalizability of the findings to other populations, such as older adults, individuals with comorbidities, or people from different cultural and ethnic backgrounds. In addition, the requirement that participants be capable of performing cycling exercise at 90% HR_max may have resulted in the inclusion of relatively fitter individuals with obesity. Therefore, the generalizability of the findings to individuals with lower fitness levels should also be interpreted with caution. Future studies are encouraged to recruit larger, more diverse samples and consider stratifying participants by fitness level to enhance ecological validity and external applicability.

## 5 Conclusion

1. High-intensity interval exercise (HIIE) may improve behavioral response speed in obese males by promoting non-inhibitory functional optimization of the prefrontal–striatal pathway, rather than engaging traditional inhibitory control systems. This effect reflects the characteristic mechanism of short-term exercise described by the neural efficiency hypothesis.
2. Moderate-intensity continuous training (MIAE) may enhance conflict monitoring capacity in obese females, facilitating a shift in inhibitory control over high-calorie food stimuli from effortful, active suppression to more automatic regulation. These findings underscore the need for food-related inhibitory interventions in obesity to consider both exercise intensity adaptability and sex-specific neuro-metabolic targets, thereby providing a scientifically grounded basis for personalized exercise prescription.

## Data Availability

The original contributions presented in the study are included in the article/Supplementary material, further inquiries can be directed to the corresponding author.
